# Barriers to early diagnosis of cervical cancer: a mixed-method study in Côte d’Ivoire, West Africa

**DOI:** 10.1186/s12905-023-02264-9

**Published:** 2023-03-27

**Authors:** Marie K. Plaisy, Simon P. Boni, Patrick A. Coffie, Aristophane Tanon, Adoubi Innocent, Apollinaire Horo, François Dabis, Anne Bekelynck, Antoine Jaquet, for the IeDEA West Africa Collaboration

**Affiliations:** 1 Research Institute for Sustainable Development (IRD) EMR 271, University of Bordeaux, National Institute for Health and Medical Research (INSERM) UMR 1219, Bordeaux Population Health Centre, Bordeaux, France; 2 National Cancer Control Program, Abidjan, Côte d’Ivoire; 3 PACCI Program, National Agency for Scientific Research (ANRS) site in Côte d’Ivoire, Abidjan, Côte d’Ivoire; 4 Tropical and Infectious Diseases Department, University Hospital of Treichville, Abidjan, Côte d’Ivoire; 5 Oncology Department, University Hospital of Treichville, Abidjan, Côte d’Ivoire; 6 Gyneco-Obstetrics Department, University Hospital of Yopougon, Abidjan, Côte d’Ivoire

**Keywords:** Cervical cancer, Advanced cervical cancer stages, Limited resources, Andersen model, Côte d’Ivoire, Mixed methods study

## Abstract

**Background:**

Cervical cancer, a major public health problem in many developing countries, is usually associated with a poor survival related to an advanced disease at diagnosis. In Côte d’Ivoire and other developing countries with high cervical cancer prevalence, little is known about factors associated with advanced cervical cancer stages in a context of limited access to screening services.

**Methods:**

From May to July 2019, we conducted a cross-sectional study using a mixed, quantitative and qualitative method. Information on socio-demographic and history of the disease was extracted from a rapid case ascertainement study performed by the cancer registry of Côte d’Ivoire that enrolled all women diagnosed with cervical cancer between July 2018 and June 2019. In-depth semi-structured interviews were conducted among a subset of these women (12 women) and six healthcare providers to further capture barriers to early cervical cancer diagnosis. Factors associated with an advanced stage III, IV (according to FIGO classification) were estimated by a logistic regression model. Qualitative data were analyzed using a thematic analysis technique guided by the treatment pathway model and triangulated with quantitative data.

**Results:**

In total, 95 women with cervical cancer [median age = 51 (IQR 42–59)] years, were included. Among them, 18.9% were living with HIV and only 9.5% were covered by a health insurance. The majority (71.5%) were diagnosed with advanced cervical cancer. Being HIV-uninfected (aOR = 5.4; [1.6–17.8], p = 0.006) and being uninsured (aOR = 13.1; [2.0-85.5], p = 0.007) were independently associated with advanced cervical cancer in multivariable analysis. Qualitative data raised additional factors potentially related to advanced cervical cancer stages at diagnosis, including the lack of patient information on cervical cancer by healthcare providers and inadequate national awareness and screening campaigns.

**Conclusion:**

In a context of challenges in access to systematic cervical cancer screening in Côte d’Ivoire, access to health insurance or integrated healthcare program appear to be key determinants of early diagnosis of cervical cancer.

## Introduction

The management of cervical cancer (CC) remains a major public health issue in developing countries, mainly due to advanced stage of the disease at diagnosis [1]. Effective prevention strategies including vaccination against human papilloma virus (HPV), CC screening followed by treatment of pre-cancerous lesions are intended to reduce incidence and mortality of CC [2–6]. Although the effectiveness and efficiency of preventive measures of CC have been widely demonstrated [7], access to HPV vaccination and screening at population-wide level remains limited in developing countries, and most CC are diagnosed at an advanced stage [8–10]. According to the latest world cancer statistics, CC is the fourth most common diagnosed cancer and the fourth leading cause of cancer death in women worldwide, with approximately 604,000 new cases and 342,000 deaths. Sub-Saharan Africa accounts for the highest incidence and mortality, partly due to the lack of formalized screening programs [11].

In Côte d’Ivoire, CC is the second most common cancer in women and the leading cause of cancer death in women. Age-standardized incidence and mortality rates are estimated at 31.2 cases and 22.8 deaths per 100,000 women in 2020, respectively [12]. Since 2009, the Ivorian Ministry of Health, through its National Cancer Control Program (PNLCa), has implemented a national program for CC screening and subsequent treatment of precancerous lesions, which are based on visual inspection with acetic acid and cryotherapy, respectively. This screening program has been subsequently scaled-up over 134 healthcare facilities. Nevertheless, as reported by a previous study, screening coverage for CC remains relatively low with a rate of only 1.2% in the urban area of Abidjan; and aproximately 70% of CC were diagnosed at an advanced stage [13, 14]. This is a serious concern, given the fact that CC is highly preventable and curable if detected and treated in the earliest stage [2]. Of note, in high-income countries, organised and comprehensive approaches to cervical screening and prophylactic treatment have significantly reduced the incidence and mortality of CC over the past decades [15–17].

Early diagnosis of CC is of far greater prognostic importance than any attempts to treat the disease at an advanced stage, as at this stage, treatment is more challenging and the risk of early cancer-related mortality is increased [18, 19]. Beyond the WHO call for elimination targeting 90% of women diagnosed with CC accessing care, access to an effective treatment is therefore largely mediated by the extension of the disease when diagnosed [20]. Accordingly, it is crucial to have a better understanding of factors associated with advanced stages of CC considering the limited information from low and middle-income countries [8, 9, 21–23]. The aim of this study was to determine the barriers to early diagnosis of CC in Côte d’Ivoire.

## Materials and methods

### Population and design

A cross-sectional study using a mixed quantitative and qualitative method was conducted in Abidjan, the economic capital of Côte d’Ivoire from July 2018 to June 2019. Our study population was nested in a larger project supported by the cancer registry of Abidjan that initiated a rapid case ascertainment of all CC diagnosed in the urban area of Abidjan during a two-year period. During this period, all units potentially managing CC from the public sector (four units from the three public referral hospitals in Abidjan and the recently opened radiotherapy center), as well as one of the major clinics from the private sector with the capacity to manage CC in the urban area of Abidjan, were asked to systematically include all adult women aged ≥ 18 years old with a suspected or confirmed diagnosis of CC. Cervical biopsies and histologic examination by the local pathology unit were systematically proposed and financially supported by the research project, when appropriate. For the purpose of the study, we included all women diagnosed with CC and being alive during the study period. Women who where unable to participate due to an impaired physical health condition, or who refused to participate in our study or could not be reached by telephone during our study period were excluded.

### Collected information

Trained clinical monitors administered a structured questionnaire to women enrolled in this research project to collect clinical and sociodemographic characteristics such as age, formal education (categorized as no school, primary school, secondary school and above), parity, personal monthly income, personal health insurance coverage. Clinical stage of cancer at CC diagnosis was assessed based on the International Federation of Gynecology and Obstetrics (FIGO) staging system [24]. Based on available information after the initial assessment of the tumor extension, clinical stage at diagnosis was reported by clinicians and dichotomized as early (stage I and II) or advanced (stage III and IV) disease.

A nationally approved rapid HIV test (Determine, Abbott Diagnosis) was systematically performed by collecting capillary blood through a finger prick during the interview. In case of positive result, a venous blood sample was collected for confirmation purposes in accordance with the national algorithm of Côte d’Ivoire.

From May to July 2019, all women included in this rapid case ascertainment study were systematically contacted by phone to obtain additional information as part of the present study. From all participants who consented to participate in the study, a sample of 12 women was selected for semi-structured interviews. They were selected purposively based on different characteristics such as age, HIV status, place of residence, International Federation of Gynaecology Obstetrics (FIGO) stage and level of education to obtain a diversified profile that reflects the caracteristics of the overall enrolled participants. The number was determined on the principle of data saturation. Additional interviews were conducted with Key Informants (KIs), including health professionals (n = 5) and public stakeholders (n = 1) worked at the participating cancer referral hospitals.

We administrated a standardized questionnaire face-to-face at the referral hospital where the patients were diagnosed for CC, and by phone for participants who could not attend in person to collect data as part of the quantitative component of the study. With regard to the qualitative component, all semi-structured interview were carried out face-to-face using an interview guide.

**Fig. 1:**
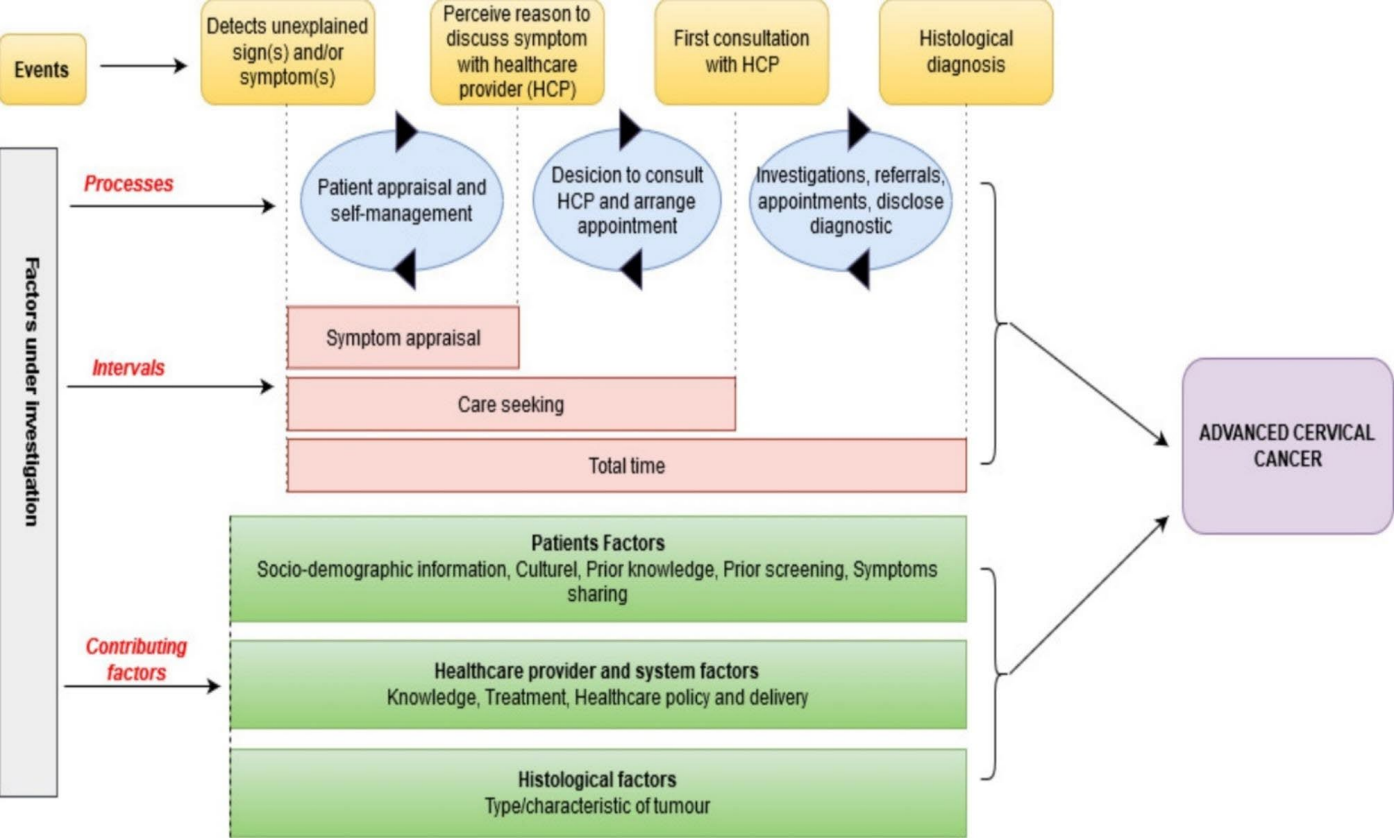
Conceptual framework of the study based on Andersen model and data from the literature

The questionnaire was designed based on the Andersen’s model (Fig. 1) and findings from studies on factors contributing to advanced CC stages in low and middle-income countries [9, 10, 22, 23, 25]. The Andersen’s model was designed to describe the decision and behavioural processes that occur prior to the diagnosis and treatment of cancer and other chronic conditions [25]. This model was adapted and we considered the intervals for symptom appraisal, care seeking and total time that can influence the diagnosis of CC. The questionnaire included questions on socio-demographics, health insurance coverage, access to traditional healers, access to specialized facilities, prior knowledge of CC, past history of cervical screening, history of the disease including the intervals based on Andersen’s model (symptom appraisal, care seeking and total time) and the pre-referral consultations. The symptom appraisal interval is defined as the time between detection of unexplained symptoms and the decision to consult a healthcare provider. The care seeking interval is the time between the first symptoms dectected and the first consultation to a healthcare provider. The total time interval represents the time between the first symptoms and the histological diagnosis of CC [25–27]. (See Fig. 1). Pre-referral consultations is defined as any visit to a healthcare provider before the diagnosis of CC [28].

The qualitative interview guide included questions related to gynecological follow-up prior to CC diagnosis, therapeutic pathway leading to CC diagnosis, various obstacles to seeking care and getting a diagnosis, cultural and religious beliefs regarding CC, cervical screening and diagnosis services in Côte d’Ivoire, with suggestions to improve the quality of such services. Time taken for each interview was about 30 to 50 min, and interviews were recorded using a dictaphone.

### Mixed methods data analysis

In the quantitative analysis, continuous variables were presented as median with their interquatile range (IQR) and categorical variables as absolute numbers and percentages (%). The measured outcome was CC stage at diagnosis dichotomised as early (stage I and II) or advanced (stage III and IV). To determine factors associated with advanced CC stages, univariable and multivariable logistic regressions were performed. Variables found be associated (p value ≤ 0.20) with advanced CC stages in univariable models were entered into the initial multvariable model. A stepwise descending procedure was performed to obtain the final model. Both unadjusted (UOR) and adjusted Odd’s ratios (AOR) were computed together with their 95% confidence limits (95% CI). A p-value of ≤ 0.05 was considered significant in the final model. The variable Knowledge of CC was not significant but remained in the model as a confounding variable for HIV status and Health insurance covariates. All statistical analysis were performed using the Version 3.5.0 of the R software.

For the qualitative component, all interviews were manually transcribed from Microsoft Word the day of the interview. Data were analyzed using thematic analysis technique with the aid of QDA miner Lite software. Guided by the Model of Pathways to Treatment, themes and sub themes have been identified, indexed, interrelated and described. These were then colour-coded, quotations were selected to represent each emerging theme and are reported as the study results [29, 30]. We further triangulated qualitative themes and quantitative data to identify areas of convergence and divergence related to factors contributing to an advanced CC stages at diagnosis. In the results section of the manuscript, verbatim quotes from participants were identified by numbers, participant age in year, and FIGO stage. However, information from Keys Informants served to guide the interpretation and discussion of our findings.

## Results

### Respondents’ characteristics

A total of 158 women with a histological diagnosis of CC between July 2018 and June 2019 were identified by the Abidjan cancer registry and requested through phone calls to participate in our study. Of these, 50 died before the period of our survey. Among the 108 alive, 13 were excluded for the following reasons: unreachable by phone (n = 6), refusal (n = 3), unaware of their disease (n = 1) or FIGO not available in the medical records (n = 3). Thus, 95 women were included in our quantitative analyses.

Their median age was 51 (IQR 42–59) years. About two third 61 (64.2%) were unmarried; 41 (43.2%) lived in Abidjan; 33 (34.7%) had no monthly income and 54 (56.8%) had monthly income less than 250 USD. Forty four women (46.0%) reported no formal education; 39 (41.1%) were unemployed and 55 (57.9%) were diagnosed with CC six months or more after the onset of the first symptoms. HIV infection was found in 18 (18.9%) respondents and only 9 (9.5%) were covered by a health insurance. Approximately 62 (65.3%) of the participants in our study had three or more consultations prior CC confirmation. All participants in our study were diagnosed with CC based on the occurrence of pre-exisiting signs and symptoms. An advanced CC stage at diagnosis was reported in 68 (71.6%) respondents and majority of them had squamous cell carcinomas 86 (90%) (Table 1).

**Table 1:** Characteristics of 95 women diagnosed with cervical cancer in Côte d’Ivoire, 2019

Characteristics	Early stage (n = 27)		Advanced stage (n = 68)		Total (n = 95)	
	n	%	n	%	n	%
***Age, median [IQR]***	49	[41–57]	52	[42–60]	51	[42–59]
***Age group (years)***						
<40	5	18.5	12	17.6	17	17.9
40–59	18	66.7	37	54.4	55	57.9
≥60	4	14.8	19	27.9	23	24.2
***Education level***						
None	11	40.7	33	48.5	44	46.3
Primary	8	29.6	20	29.4	28	29.5
Secondary and over	8	29.6	15	22.0	23	24.2
***Marital status***						
Married	12	44.4	22	32.4	34	35.8
Widowed	5	18.5	20	29.4	25	26.4
Living with partner	6	22.2	12	17.6	18	18.9
Single or divorced	4	14.8	14	20.6	18	18.9
***Occupation***						
Employed	17	63.0	39	57.4	56	58.9
Unemployed	10	37.0	29	42.6	39	41.1
***Number of biological children***						
< 5	12	44.4	31	45.6	43	45.3
≥5	15	55.6	37	54.4	52	54.7
***HIV status***						
Positive	9	33.3	9	13.2	18	18.9
Negative	18	66.7	59	86.8	77	81.1
***Place of living***						
Abidjan	10	37.0	31	45.6	41	43.2
Other cities^$^	17	63.0	37	54.4	54	56.8
***Health Insurance coverage***						
Insured	6	22.2	3	4.4	9	9.5
Uninsured	21	77.8	65	95.6	86	90.5
***Monthly income (USD)***						
None	8	29.6	25	36.8	33	34.7
<250	14	51.9	40	58.8	54	56.8
≥250	5	18.5	3	4.4	8	8.4
***Estimated distance from home*** ***to referral hospital (Kilometers)***						
< 100	12	44.4	36	52.9	48	50.5
≥ 100	15	55.6	12	47.1	47	49.5
***Histological subtypes***						
Adenocarcinoma	1	3.7	8	11.8	9	9.5
Squamous cell carcinoma	26	96.3	60	88.2	86	90.5
***Total time interval*** *® (days)*						
Median, [IQR]	231	[120–379]	208	[113–439]	216	[117–429]
< 180	10	37.0	30	46.1	40	42.1
≥ 180	17	63.0	38	53.9	55	57.9
***Number of pre-diagnosis visits***						
Median, [IQR]	3	[2–4]	3	[2–4]	3	[2–4]
<3	9	33.3	24	35.3	33	34.7
≥3	18	66.7	44	64.7	62	65.3

Notes: ®, time between the first symptoms and the histological diagnosis of cancer; $, people who lived in other districts of Côte d’Ivoire except Abidjan

A total of 18 semi-structured interviews were conducted as part of the qualitative component; 12 with women diagnosed with CC and 6 with key informants (KIs), i.e. health professionals and public stakeholders. Most of the women involved in the qualitative survey lived in Abidjan (8/12). Half of them reported no formal education and low economic level (those with a monthly income of less than 250 $USD). Four of them were infected with HIV at the time of CC diagnosis and 8 were diagnosed with advanced CC (FIGO III or IV). (Table 2). All KIs respondents were working at health facilities in Abidjan. Among them, two were oncologists of which one also worked at the PNLCA, two KI were gynaecologists and practiced respectively at the university hospital and general hospital, and two were midwives. (Table 3).

**Table 2:** Characteristics of women interviewed in the qualitative component, Côte d’Ivoire, 2019

Participant	Age (years)	City of residence	Education level	Socio-Economic Status	HIV Status	Health Insurance	FIGO stage
P1	59	Abidjan	Primary	Low	Negative	Uninsured	III
P2	38	Lagunes	None	Low	Negative	Uninsured	IV
P3	54	Abidjan	None	Very Low	Positive	Uninsured	IV
P4	34	Abidjan	None	Very Low	Negative	Uninsured	III
P5	56	Zanzan	None	Low	Negative	Uninsured	III
P6	38	Abidjan	None	Low	Positive	Insured	I
P7	36	Abidjan	None	Very Low	Negative	Uninsured	III
P8	43	Bas-Sassandra	Primary	Low	Negative	Uninsured	III
P9	43	Abidjan	Primary	Low	Positive	Uninsured	II
P10	49	Comoé	Primary	Very Low	Positive	Uninsured	II
P11	38	Abidjan	Secondary and over	Medium	Negative	Insured	II
P12	49	Abidjan	Secondary and over	Medium	Negative	Uninsured	IV

Abbreviations: FIGO- International Federation of Gynaecology and Obstetrics; P- Participant

Notes: Very low socio-economic status- Participant with no monthly income, Low socio-economic status- Participant with a monthly income less than 250 USD, Medium socio-economic status- Participant with a monthly income between 250 USD and 500 USD

**Table 3:** Characteristics of Keys informants interviewed in the qualitative component, Côte d’Ivoire, 2019

Characteristics	KI (1)	KI (2)	KI (3)	KI (4)	KI (5)	KI (6)
Age (years)	48	44	59	36	45	49
Gender	W	M	W	M	M	M
Designation	Midwives	Oncologist	Midwives	Gyneacologist	Gyneacologist	Oncologist
Working experience (years)	10	9	22	9	15	11

Notes: W- Women; M- Men; KI- Key Informant

### Factors associated with advanced cervical cancer

#### Patient related factors

In the multivariable model, HIV-uninfected respondents were more likely to have advanced CC than those HIV-infected (AOR = 5.4; 95% CI [1.6–17.8], p = 0.006). (Table 4).

**Table 4:** Factors associated with advanced cervical cancer (FIGO stage: III or IV), Côte d’Ivoire, 2019, (n = 95)

	Cancer Stage at diagnosis					
	Early stage (I/II)	Advanced stage (III/IV)	Unadjusted OR (95% CI)	P value	Adjusted OR (95% CI)	P value
**Factors**	n/N	n/N				
***Age group (years)***				0.36		
<40	5/17	12/17	1			
40–59	18/55	37/55	0.8 [0.2–2.8]			
≥60	4/23	19/23	2.0 [0.4–8.9]			
***Education level***						
None	11/44	33/44	1	0.70		
Primary	8/28	20/28	0.8 [0.3–2.4]			
Secondary and over	8/23	15/23	0.6 [0.2–1.9]			
***Marital status***				0.32		
Married	12/34	22/34	1			
Living with partner	6/18	12/18	1.1 [0.3–3.6]			
Widowed or single or divorced	9/43	34/43	2.0 [0.7–5.7]			
***Occupation***						
Employed	17/56	39/56	1			
Unemployed	10/39	29/39	1.2 [0.51–3.16]	0.61		
***Place of living***						
Abidjan	10/41	31/41	1			
Other cities^**$**^	17/54	37/54	0.7 [0.3–1.7]	0.44		
***Menopause***						
No	15/36	21/36	1			
Yes	12/59	44/59	2.8 [1.1–7.0]	**0.02**	-	-
***HIV Status***						
Positive	9/18	9/18	1		1	
Negative	18/77	59/77	3.3 [1.1–9.5]	**0.02**	5.4 [1.6–17.7]	**0.006**
***Health insurance coverage***						
Yes	6/9	3/9	1		1	
No	21/86	65/86	6.2 [1.4–26.9]	**0.01**	13.1[2.0–85.5]	**0.007**
***Prior Knowledge of cervical cancer™***						
Yes	19/51	32/51	1		1	
No	8/44	36/44	2.7 [1.0–6.9]	**0.04**	2.1 [0.7–6.3]	0.16*
***Use traditional healers*** *©*						
No	22/63	51/63	1			
Yes	5/32	27/32	2.9 [1.0–8.5]	**0.05**	-	-
***Access to specialized facilities*** *¥*						
Reference	25/76	51/76	1		1	
Directly	2/19	17/19	4.2 [0.9–19.4]	**0.07**	7.1 [1.1–44.0]	**0.01**
***Number of pre-diagnosis visits***						
<3	9/33	24/33	1			
≥3	18/62	44/62	0.9 [0.3–2.3]	0.85		
***Histological subtypes***						
Adenocarcinoma	1/9	8/9	1			
Squamous cell carcinoma	26/86	60/86	0.3 [0.03–2.4]	0.25		
***Total time interval*** *®*						
<180	10/40	30/40	1			
≥180	17/55	38/55	0.7 [0.3–1.8]	0.52		

Bold typeface indicates statistically significant values

*Confounding factor with HIV status and Health insurance coverage

Notes: $, people who lived in other districts of Côte d’Ivoire except Abidjan; ™, Prior knowledge of CC before disease onset; ©, Use of traditionals healthworkers after recognition of early symptoms; ¥, How women get access to specialized facilities; ®, time between the first symptoms and the histological diagnosis of cancer.

Abbreviations: OR- Odds ratio; CI- Confidence Interval

In-depth interviews showed that it is not usual for healthy ivorian women to have regular gynaecological follow-up. However, patients with a chronic disease, such as HIV, are more likely to be tested early within the management of their disease according to national guidelines.

*“... I used to get tested for cervical cancer every year at the HIV clinic where I received my medication. The last time was in 2017.“* (P6, 38 years old, stage I)

*”Before the illness, I didn’t use to consult the gynaecologist because I had no disease.“* (P2, 38 years old, stage IV).

*“Africans do not visit gynaecologist except for childbirth or other serious health problems. And often, in our culture, we say to avoid waking up the sleeping illness. Laughing.“* (P8, 43 years old, stage III)

Being uninsured was significantly associated with advanced CC (AOR = 13.1, 95% CI [2.0-85.5], p = 0.007) (Table 4). In-depth interviews, lack of money was mentioned by most respondents as a major cause of delay in performing the test required to confirm the diagnosis of CC. One participant mentioned that she was privileged to perform the biopsy early and to be diagnosed at an early stage of the disease through her health insurance.

“*Biopsy is still expensive. I was lucky enough to have it done the same day it was prescribed and to be diagnosed at an early stage because my insurance covered more than half of the costs.”* (P11, 38 years old, stage II)

*“I couldn’t do the biopsy the same day it was prescribed because of lack of money. So I waited two weeks before coming back to do it for free at the university hospital.”* (P7, 36 years old, stage III)

Participants who attended directly to specialized facilities were more likely to be diagnosed with advanced CC compared to those who had been referred by peripheral healthcare providers (AOR = 7.05, 95% CI [1.13–44.07], p = 0.03).

During our in-depth interviews, some participants reported that the main reason they attended specialized facilities directly was related to the deterioration of their health conditions as they were not familiar with CC and its treatment when the disease first appeared.

*“At the beginning, I didn’t consider my illness, I thought it was common. It was a few months later, when I saw that I was bleeding too much and becoming very weakened, I went directly to the university hospital.”* (P1, 59 years old, stage III)

Statistically significant association was not found between the total number of pre-diagnosis visits (OR = 0.9, 95% CI [0.3–2.3], p = 0.85), total time interval (OR = 0.7, 95% CI [0.3–1.8], p = 0.52, histologic type (OR = 0.3, 95% CI [0.0-2.4], p = 0.25) and advanced CC stages. (Table 4).

#### Health providers-related factors

In-depth interviews highlighted the role of healthcare providers in the diagnosis of advanced CC stages. Two sub-themes were identified as priorities: lack of patient information and awareness about CC, and inadequate knowledge on signs and symptoms of CC.

#### 1. Lack of patient information and awareness about cervical cancer and screening by healthcare providers

Some participants reported that the physicians they had consulted prior to the beginning of their disease were partly responsible for the advanced stage of their cancer. They mentioned that they had never been informed about CC, or been proposed the screening test during their previous medical visits. For others participants, while information about CC was provided and CC screening was proposed by physicians, the information appeared to be unclear and not explicit. During the interview, a respondent mentioned not understanding the benefits of the test, nor the gravity of the disease and, therefore did not return for the follow-up. This would have contributed to poor uptake or adherence to the screening test, leading to advanced stages of CC at diagnosis.

*“If I went to the gynaecologist before my illness, it was also to orient me and help me avoid other illnesses. He prescribed blood tests and echography, but he never told me about cervical cancer and did not proposed me to do a pap test.”* (P12, 49 years old, stage IV).

*“The doctor I was following asked me to do this test every year. Each time I was tested, he never gave me the result. I didn’t even know why I was doing it… For the past four years, I have not returned for this test.”* (P3, 54 years old, stage IV)

#### 2. Inadequate knowledge of the signs and symptoms of cervical cancer

Some respondents mentioned that some healthcare providers appear to be unfamiliar with the signs and symptoms of CC. This sometimes leads to misdiagnosis and inappropriate management, which can be a barrier to early diagnosis of CC.

*“At the General Hospital, the doctor who examined me gave me a blood test, then told me that I had malaria.”* (P5, 56 years old, stage III)

*“[...] As the bleeding persisted, I went to see a midwife at the general hospital. After examination, she told me that my cervix was open. If at least she knew the small signs of cervical cancer, she would have referred me immediately to the specialized hospital to see a gynecologist.”* (P7, 36 years old, stage III)

*“[...] When I arrived at the dispensary, the nurse gave me two injections to stop the blood, then asked me to come back a week later. I didn’t return because the injections were very painful and worsening my condition.”* (P5, 56 years old, stage III)

### Health system factors

#### Inadequate national awareness and screening campaigns

Inadequate national awareness and screening campaigns were mentioned as a barrier to early diagnosis of CC, as exemplified by the women in our study. Some women elaborated the poor distribution of these campaigns, which are more concentrated in large cities than in villages. They also reported the irregularity of these campaigns, the ambiguity of the information provided, and the lack of CC awareness campaigns compared to other diseases. The unsuitability of communication tools within the local context was also emphasized by some respondents.

*“[…] There is more awareness campaigns on breast cancer than cervical cancer. Those who talk about cervical cancer don’t give enough information about its causes and symptoms.”* (P12, 49 years old, stage IV)

*“I have heard that the message about cervical cancer and screening is played only on the radio. However, in my village, I don’t have a radio. That’s why I didn’t have access to information about it before my illness.”* (P4, 34 years old, stage III)

## Discussion

To the best of our knowledge, this is the first study conducted in Côte d’Ivoire to identify barriers to early diagnosis of CC. Our findings showed that some characteristics related to patients (HIV-uninfected, being uninsured), healthcare providers (inadequate knowledge of the signs and symptoms of CC, lack of patient information and awareness about CC), and the health system (inadequate awareness and screening campaigns) were higlighted as potential drivers of advanced CC stages at diagnosis.

In our study, more than two-thirds of respondents were diagnosed with advanced CC. This finding is consistent with other studies conducted in Sudan (71.5%) [31] and Abidjan (70.0%) [13]. Based on the litterature, CC diagnosed at an advanced stage is associated with adverse complications, poor treatment outcomes, and high mortality rate [18, 32]. This study showed that women who did not have medical insurance were more likely to be diagnosed at an advanced CC stages than those who had medical insurance. Similar finding was also reported in Sudan, showing a correlation between lack of health insurance and advanced CC (OR = 7.7; [3.76–15.38]) [31]. Based on our findings, lack of health insurance can hinder some ivorian women to attend the hospital for a medical check-up or in case of illness, and to afford the medical fees. In Côte d’Ivoire, the overall costs for CC diagnosis, including consultation and biopsy fees, is quite expensive. As reported by the PNLCa, patients could pay between 34 and 37 $USD in the public sector; and between 59 and 76 $USD in the private sector. Understandably, this could be a financial barrier for most ivorian women and could delay the realization of the biopsy to confirm the CC. Our study revealed that few of the participants were covered by health insurance. Access to universal health coverage in Côte d’Ivoire might reduce social health inequalities, advanced CC stages and others chronic conditions, and address the health needs of the ivorian population. Several studies have shown the positive effect of health insurance on the early diagnosis of some cancers (breast, colon, lung, uterus, prostate) [33–35]. In line with others studies, HIV-infected participants were diagnosed with CC at an earlier stage than did their uninfected counterparts. This statement is consistent with results of two studies performed in Uganda and Bostwana [36, 37]. It is mainly related to the lack of access to healthcare and/or poor services especially in public facilities in limited-income countries. Because of inadequate access to healthcare, women in the general population have no incentive to perform gynaecological follow-up and consult a physician at the onset of signs and symptoms for an early diagnosis. On the other hand, HIV-infected women are more likely to be diagnosed at an early stage, as CC screening has been increasingly integrated into the package of services for HIV-infected women over the past decade, ensuring a better protection towards advanced stages. In line of this finding, integration of CC screening programs into other pre-existing care systems could improve access and quality of care, by providing a holistic care to all patients attending the health facility [38–40].

Results of our study showed that participants who came directly to specialized facilities were more likely to be diagnosed at an advanced stage than those who had been quickly referred by peripheral healthcare providers. This is considered as a consequence of the advanced stage of CC, as most of the women who came directly to a specialized facility presented a deteriorating conditions, and most of them were diagnosed at an advanced stage. This finding could be explained by women’s limited knowledge and awareness about the signs and symptoms of CC and/or inadequate management of their symptoms during the previous visits. Furthermore, results of our qualitative approach suggest that inadequate knowledge of the signs and symptoms of CC, especially among primary healthcare providers, leading to misdiagnosis and inappropriate management. This statement is consistent with results from two studies conducted in Rwanda and Malawi [23, 41]. Aside from the gap in CC knowledge, some healthcare providers do not inform their patients about CC and the screening procedures. This may reflect the limited effectiveness of the CC screening programs implemented by the ivorian ministry of health that have so far. Lack of knowledge and awarness towards CC screening surely contributes to the under-use of CC screening services, a key factor in the prevention of advanced CC diagnosis. Indeed, a few of the participants in our study had been screened for CC prior to their illness. Previous studies in Ghana and Iran have demonstrated a significant association between lack of CC screening and advanced CC stage [8, 42].

These findings showed that significant efforts through the implementation of early CC surveillance programs, including public awareness and health providers’ education about the disease could reduce the proportion of advanced CC. Accordingly, results of a pilot program in Malaysia, which focused on women’s and health professionals’ education, showed a significant reduction in advanced cervical and breast cancers, along with a significant cost-effectiveness [43]. Results from others studies have shown that large-scale interventions, including public awarness and education, have reduced the proportion of cancer diagnosed at an advanced stage [44, 45].

Our quantitative results revealed no association between total time interval prior CC diagnosis and advanced CC. The same result was found in a study conducted in northern Uganda [22]. All participants in our study were diagnosed with CC based on pre-exisiting signs and symptoms. Most of them were at an advanced stage at the time of recognition of these signs and symptoms as abnormal, regardless of the number of pre-diagnosis visits and the total time interval. These results confirmed that access to a systematic CC screening remains a key determinant to reduce the proportion of advanced CC, as mentioned above.

The current study has some limitations to consider. A significant number of women initially enrolled in the cancer registry project had died by the time our survey was conducted. Moreover, some suspected cases identified outside of Abidjan could not reach the town to confirm the diagnosis of CC through biopsy. This could potentially underestimate the proportion of CC diagnosed at an advanced stage. However, the proportion of women with advanced CC is quite similar to findings from previous studies in Côte d’Ivoire and sub-Saharan African countries [13, 22, 31]. Retrospective recall bias could have been introduced when determining the exact time intervals between the onset of symptoms and the diagnosis. It was often difficult for participants to accurately determine the date of the onset of their symptom, or to recognize whether the symptoms were actually associated with CC. However, in such a mixed-methods study, the inability to accurately determine the intervals of symptom onset, care-seeking, and total time could not affect participants’ perceptions of the therapeutic pathway leading to CC diagnosis.

## Conclusion

Factors facilitating access to healthcare system prior to the diagnosis of CC, including being insured, availability of a vertical healthcare program as HIV, tend to be protective factors for diagnosis of advanced CC in Côte d’Ivoire. Qualitative interviews with women diagnosed with CC and key informants revealed several gaps in knowledge about CC and its prevention. Further studies involving primary care workers are needed to confirm their potential role in the diagnosis of advanced CC. Improvement of the existing education and awareness raising programs targeting women and healthcare providers should also be considered, and its impact on advanced CC stages should be explored.

In addition, large-scale awareness and education campaigns are needed to promote access to systematic CC screening and to improve the ongoing training of healthcare providers, especially primary care providers, to reduce advanced CC stages. Moreover, findings of this study highlight the urgent need to implement an inclusive health insurance system which contributes to the early detection of CC and the reduction of health inequalities towards the elimination of CC, a WHO target by 2030.

## Data Availability

The datasets generated and/or analyzed during the current study are not publicly available as they contain confidential information that could compromise privacy but can be made available with non-identifiable aspects from the corresponding author on reasonable request.

## Declarations

### Disclaimer

The content is solely the responsibility of the authors and does not represent the official views of The National Cancer Institute and the National Institutes of Health.

### The IeDEA West Africa Collaboration

Désiré Dahourou^7^, Sophie Desmonde^7^, Julie Jesson^7^, Valeriane Leroy^7^, Antoine Jaquet^1^, Didier Koumavi Ekouevi^1^, François Dabis^1^, Renaud Becquet^1^, Charlotte Bernard^1^, Karen Malateste^1^, Olivier Marcy^1^, Marie Kerbie Plaisy^1^, Elodie Rabourdin^1^, Thierry Tiendrebeogo^1^, Raoul Moh^3^, Jean-Claude Azani^3^, Jean Jacques Koffi^3^, Eric Komena^3^, Maika Bengali^3^, Abdoulaye Cissé^3^, Guy Gnepa^3^, Apollinaire Horo^6^, Simon Boni^2^, Eulalie Kangah^3^, Corinne Moh^3^, Jeanne Eliam^3^, Ighovwerha Ofotokun^8^, Noelle Benzekri^9^, Geoffrey Goettlieb^9^, Marcel Djimon Zannou^10^, Armel Poda^11^, Oliver Ezechi^12^, Eugene Messou^13^, Henri Chenal^14^, Kla Albert Minga^15^, Aristophane Tanon^4^, Moussa Seydi^16^, Ephrem Mensah^17^, Caroline Yonaba^18^, Lehila Bagnan Tossa^10^, Marcelline d’Almeida^10^, Jocelyn Dame^19^, Sylvie Marie N’Gbeche^13^, Kouadio Kouakou^14^, Madeleine Amorissani Folquet^20^, François Tanoh Eboua^21^, Fatoumata Dicko Traore^22^, Agatha David^12^, Elom Takassi^23^.

**Affiliations**:

^7^Inserm UMR 1295, Toulouse, France.

^8^Emory University, Atlanta, USA.

^9^ Washington University, Washington, USA.

^10^CNHU, Cotonou, Benin.

^11^CHU Souro Sanou, Bobo Dioulasso, Burkina Faso.

^12^National Institute of Medical Research (NIMR), Lagos, Nigeria.

^13^ACONDA CePReF, Abidjan, Côte d’Ivoire.

^14^CIRBA, Abidjan, Côte d’Ivoire.

^15^CMSDS, Abidjan, Côte d’Ivoire.

^16^CHU de Fann, Dakar, Senegal.

^17^Clinique EVT, Lomé, Togo.

^18^CHU Yalgado Ouédraogo, Ouagadougou, Burkina Faso.

^19^Korle Bu Hospital, Accra, Ghana.

^20^CHU de Cocody, Abidjan, Côte d’Ivoire.

^21^CHU de Yopougon, Abidjan, Côte d’Ivoire.

^22^Hôpital Gabriel Touré, Bamako, Mali.

^23^CHU Sylvanus Olympio, Lomé, Togo.

## List of abbreviations

AOR: Adjusted Odd’s Ratio
CC: Cervical cancer
FIGO: International Federation of Gynaecology and Obstetrics
HPVs: Human papillomaviruses
KIs: Keys Informants
PNLCA: National Cancer Control Program
OR: Odd’s Ratio.
